# Clinical characteristics of hospitalized adults and adolescents with herpes zoster in Croatia: more than 20 years of a single-center experience

**DOI:** 10.3325/cmj.2020.61.401

**Published:** 2020-10

**Authors:** Dalibor Vukelić, Dorotea Oroši Končić, Jelena Prepolec, Iva Škrabić, Andrea Šupe Parun, Tomislava Skuhala, Vladimir Trkulja

**Affiliations:** 1 Department of Infectious Diseases, University of Zagreb School of Medicine, Zagreb, Croatia; 2 Independent scholar, Zagreb, Croatia; 3 The Hospital of the Holy Spirit, Rome, Italy; 4 Community Health Centre “Zagreb – Zapad,” Zagreb, Croatia; 5 Croatian Institute of Public Health, Zagreb, Croatia; 6 Department for Urogenital Infections, University Hospital for Infectious Diseases “Dr. Fran Mihaljević,” Zagreb, Croatia; 7 Department of Pharmacology, University of Zagreb School of Medicine, Zagreb, Croatia

## Abstract

**Aim:**

To evaluate the clinical characteristics of adult and adolescent Croatian patients hospitalized for herpes zoster over a period of 21 years in the largest national center for infectious diseases (catchment area approximately 25% of the Croatian population).

**Methods:**

This retrospective chart review included all patients older than 15 years hospitalized for herpes zoster at the University Hospital for Infectious Diseases “Dr. Fran Mihaljević” between January 1, 1996 and December 31, 2016.

**Results:**

The study enrolled 1755 patients (uniform annual hospitalizations), 50% of whom suffered from complicated forms of herpes zoster, mostly generalized zoster (22.0%), infected lesions (14.8%), and meningitis/encephalitis (10.4%). A low percentage of patients experienced Ramsey-Hunt syndrome (3.0%), keratitis (1.5%), and visceral dissemination (0.2%). The majority of patients were older than 55 years (80%, median 70 years). Overall, 61.6% of patients suffered from at least one comorbidity (most frequent: diabetes 14.6%, cardiovascular incidents 24.4%, malignancy 13.0%, other infection 12.9%), and 28.2% suffered from ≥2 comorbidities. All-cause in-hospital mortality was 0.9%. The proportion of patients with any complicated form and of patients with meningitis/encephalitis steadily decreased over time, while the proportion of patients with comorbidities increased. This coincided also with steadily increasing age. No association was observed between comorbidities and complicated forms of zoster. Pharmacological immunosuppression was associated with generalized zoster; younger age was associated with meningitis/encephalitis; and older age was associated with generalized zoster and infected lesions.

**Conclusion:**

The patients most frequently hospitalized for herpes zoster are elderly people burdened with comorbidities, not necessarily patients with complicated forms of the disease.

Herpes zoster (HZ) results from a reactivation of latent varicella-zoster virus infection that gained access to sensory ganglia during the primary infection (varicella). It is clinically characterized by typical painful vesicular skin eruptions, usually occurring in a restricted dermatomal distribution (1). The prognosis of HZ is mostly favorable. However, around 14% of patients develop complications –generalized (extradermatomal) rash or infected lesions; herpetic keratitis; Ramsey-Hunt syndrome (herpes zoster oticus); herpetic meningitis/meningoencephalitis, or visceral dissemination of the infection – and 1%-4% of patients with HZ are hospitalized (2,3).

The estimated lifetime risk of HZ in the general population is around 30%, with the incidence significantly increasing after the age of 50 (1). HZ risk factors are immunosuppression, older age, and comorbidities (4-7), and annual incidence in Europe and America is estimated at 4-5 per 1000 inhabitants (8-11). There are no relevant publicly available data on HZ epidemiology in Croatia. Unpublished data from the Croatian Institute for Public Health (12) suggest doubling of the annual incidence between 1996 and 2016 from 0.4 to 1/1000 inhabitants, but this seems to be an underestimate; in the same period, the incidence in the developed European countries increased from 2 to 4-5/1000 inhabitants (9,12-16). Data on clinical presentation, co-existing factors and comorbidities, and outcomes in Croatian HZ patients are also lacking. We aimed to describe clinical disease characteristics and outcomes in adult patients and adolescents with HZ hospitalized over a period of 21 years at the largest national center for infectious diseases with a catchment population of around 1.1 million (around 25% of the total Croatian population), which also treats complex patients from the entire country.

## Patients and methods

This retrospective chart review enrolled all adults and adolescents (>15 years of age) hospitalized for HZ between January 1, 1996 and December 31, 2016 at the University Hospital for Infectious Diseases “Dr. Fran Mihaljević”, Zagreb, Croatia, a tertiary care teaching hospital affiliated with the Zagreb University School of Medicine. The study was approved by the hospital’s Ethics Committee.

### Patient identification and data extraction

For the initial patient identification, three researchers (D.O.K., J.P., I.Š.) independently searched the hospital electronic database system using ICD codes 053 (ICD-9) or B02 (ICD-10) and retrieved the archived hardcopy medical documentation. All patients were enrolled based on consensus identification. Anticipating the possibility of missing data across the records kept over 20 years, we focused on the information expected to be recorded for each patient: demographics; clinical manifestation of HZ; length of hospitalization; status at discharge; major comorbidities classified as diabetes mellitus, hypo-/hyperthyroidism, tuberculosis, any other concomitant infectious disease, chronic obstructive pulmonary disease, cardiac arrhythmia, moderate-severe liver lesion or liver cirrhosis, dementia, psychosis or psychoorganic syndrome, any neurodegenerative disease, epilepsy, stroke sequels, peripheral painful syndromes, history of cancer (solid organ or hematological), any chronic inflammatory disease, and history of major adverse cardiac events (MACE) including history of acute myocardial infarction, stroke (ischemic or transitory ischemic attack; hemorrhagic), and chronic heart failure regardless of clinical stage or venous thromboembolism within 6 months. Of the concomitant treatments, we focused on immunosuppressive therapy, ongoing or delivered within the 6 months before index hospitalization, including classical or new disease-modifying antirheumatic drugs, or any classical or new immunosuppressant used for the treatment of cancer or allergies, or after organ transplantation. We considered classical anti-cancer treatment (chemotherapy or radiation) as immunosuppressant. Individual patient data were mutually re-checked by the three researchers (D.O.K., J.P., I.Š.) and possible disagreements were resolved by a consensus. For each patient, Charlson comorbidity index was calculated. Data were entered into an electronic database and independently re-checked.

### Patient management

Patients were hospitalized by discretion of the attending physicians based on uniform institutional criteria that were in place during the observed period: complicated zoster (generalized skin lesions, infected skin lesions, or other cutaneous complications; visceral extension of dissemination; neurological complications, eg, meningitis/encephalitis, Ramsey-Hunt syndrome, ophthalmic involvement) or a simple form of the disease in patients at risk of developing complications due to, eg, comorbidity (other infections, malignancy, autoimmune diseases, severe cardiovascular diseases, limited mobility), immunosuppression, or older age. The simple form of the disease was defined as the presence of skin lesions that affected up to two adjacent dermatomes. The diagnosis of HZ in most hospitalized patients was exclusively clinical.

### Data analysis

Data on clinical presentation, patient demographics, comorbidities, hospitalization days, and clinical outcomes are summarized overall and by calendar year. Three-year rolling averages were calculated to illustrate the trends over the observed period in major disease and comorbidity features. In the exploratory analysis, we assessed potential independent associations between demographics and major comorbidity characteristics and four outcomes of interest: a) the probability of having any form of complicated disease; b) the probability of developing generalized skin lesions; c) the probability of developing infected skin lesions, and d) the probability of developing herpetic meningitis/encephalitis. Four hierarchical generalized linear mixed models (a separate model for each outcome: logit link, binary distribution, Kenward-Roger degrees of freedom, and unstructured covariance) were fitted with the same fixed effects (regardless of their actual association with the outcome): age, sex, diabetes mellitus, co-existing infection, MACE, any malignant disease, any immunosuppressive therapy, and calendar year (as a continuous variable – to evaluate trends over time; we tested linear, quadratic, and cubic trends). Calendar year (categorical) was used as a random effect to account for a possible correlation between patients hospitalized during the same year. To additionally control the type I error rate, all effects observed in each model were adjusted for multiplicity: estimates (, estimated covariance matrix [*Cov*(], and degrees of freedom were retained and used to adjust the observed confidence intervals (simulation method) and *P*-values (simulation method, logical stepdown approach). The analysis was performed with SAS 9.4 for Windows (SAS Inc., Cary, NC, USA).

## Results

### Disease and patient characteristics

**Figure 1:**
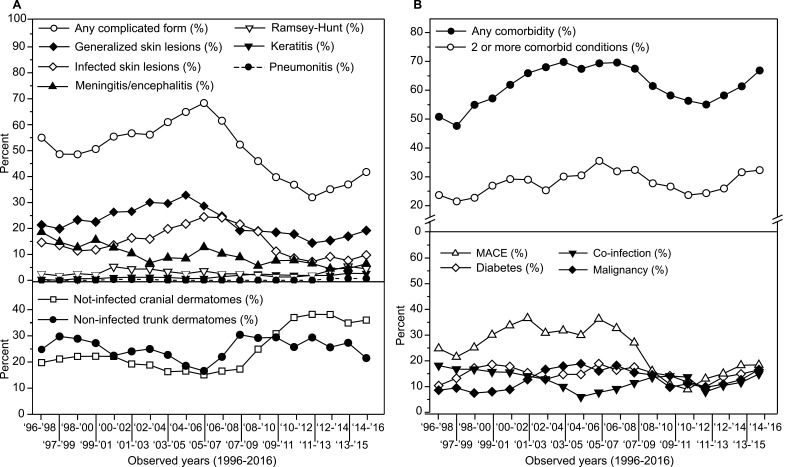
Major disease and comorbidity characteristics in hospitalized Croatian patients with herpes zoster in the period 1996-2016: 3-year rolling averages over the observed period. (**A**) Clinical presentations. (**B**) Major comorbidities. MACE – major adverse cardiovascular events

During the observed period, there were 1755 hospitalized patients, 878 (50%) of whom presented with some form of complicated HZ. The annual number of hospitalizations somewhat varied (between 67 in 2010 and 99 in 2000) but was generally constant (Table 1). The proportion of patients with a complicated HZ form also varied (Table 1), but three-year rolling averages suggested a consistent decline from 2004-2006 to the end of the observed period (Figure 1A). This coincided with a decreasing proportion of patients with infected skin lesions and meningitis/encephalitis, and a trend of somewhat lower proportions of patients with generalized skin lesions (Figure 1A). The proportion of patients with uncomplicated skin lesions increased (Figure 1A). The prevalence of patients with Ramsey-Hunt syndrome or herpetic keratitis was consistently low, and visceral dissemination was sporadic – pneumonitis was observed in only 3 (0.2%) patients (Table 1).

**Table 1:** Clinical presentations of herpes zoster in patients hospitalized during the observed period. Data are count (%)

		Any complicated		Specific complicated zoster forms					Non-infected skin lesions	
Year	N	zoster form*	generalized	infected lesions	brain^✝^	oticus	keratitis	lungs	cranial	trunk
Total	1755	878 (50.0)	386 (22.0)	259 (14.8)	182 (10.4)	53 (3.0)	26 (1.5)	3 (0.2)	442 (25.2)	435 (24.8)
1996	85	51 (60.0)	17 (20.0)	12 (14.1)	23 (27.1)	2 (2.3)	1 (1.2)	0	15 (17.6)	19 (22.4)
1997	73	43 (58.9)	11 (15.1)	15 (20.5)	17 (23.3)	2 (2.7)	0	0	17 (23.3)	13 (17.8)
1998	76	35 (46.1)	22 (28.9)	7 (9.2)	4 (5.3)	2 (2.6)	0	0	14 (18.4)	26 (34.2)
1999	83	34 (41.0)	13 (15.7)	9 (10.8)	13 (15.7)	0	0	0	18 (21.7)	31 (37.4)
2000	99	58 (58.6)	25 (25.2)	14 (14.1)	17 (17.2)	5 (5.1)	2 (2.0)	0	26 (26.3)	15 (15.2)
2001	86	45 (52.3)	23 (26.7)	9 (10.5)	12 (13.9)	1 (1.2)	0	0	16 (18.6)	25 (29.1)
2002	74	41 (55.4)	20 (27.0)	12 (16.2)	5 (6.8)	7 (9.5)	1 (1.3)	1 (1.4)	16 (21.6)	17 (23.0)
2003	85	53 (62.4)	22 (25.9)	19 (22.3)	9 (10.6)	2 (2.4)	2 (2.3)	0	15 (17.6)	17 (20.0)
2004	75	38 (50.7)	28 (37.3)	7 (9.3)	2 (2.7)	1 (1.3)	0	0	13 (17.3)	24 (32.0)
2005	93	65 (69.9)	24 (25.8)	26 (28.0)	12 (12.9)	6 (6.4)	1 (1.1)	0	13 (14.0)	15 (16.1)
2006	93	69 (74.2)	33 (35.5)	26 (28.0)	9 (9.7)	0	1 (1.1)	0	17 (18.3)	7 (7.5)
2007	69	42 (60.9)	17 (24.6)	12 (17.4)	11 (15.9)	3 (4.4)	0	0	9 (13.0)	18 (26.1)
2008	93	46 (49.5)	13 (14.0)	25 (27.0)	5 (5.4)	3 (3.2)	3 (3.2)	0	17 (18.3)	30 (32.3)
2009	73	34 (46.6)	14 (19.2)	15 (20.5)	4 (5.5)	0	2 (2.7)	0	15 (20.6)	24 (32.9)
2010	67	28 (41.8)	16 (23.9)	6 (9.0)	4 (6.0)	2 (3.0)	1 (1.5)	0	24 (35.8)	15 (22.4)
2011	97	30 (30.9)	12 (12.4)	4 (4.1)	11 (11.3)	1 (1.0)	2 (2.1)	0	35 (36.1)	32 (33.0)
2012	87	33 (37.9)	15 (17.2)	11 (12.6)	5 (5.7)	0	2 (2.3)	0	34 (39.1)	19 (21.8)
2013	81	22 (27.2)	11 (13.6)	4 (4.9)	2 (2.5)	4 (4.9)	1 (1.2)	0	32 (39.5)	27 (33.3)
2014	92	37 (40.2)	14 (15.2)	9 (9.8)	5 (5.4)	6 (6.5)	2 (2.2)	2 (2.2)	33 (35.9)	20 (21.7)
2015	85	37 (43.5)	18 (22.2)	7 (8.2)	5 (5.9)	4 (4.7)	4 (4.7)	0	25 (29.4)	23 (27.1)
2016	89	37 (41.6)	18 (20.2)	10 (11.2)	7 (7.9)	2 (2.2)	1 (1.1)	0	38 (42.7)	14 (15.7)

*One or more complicated forms: generalized skin lesions, infected skin lesions.

✝Brain affected (meningitis/meningoencephalitis), eye affected (keratitis), affected lungs, or *n. opticus* (Ramsey-Hunt syndrome).

**Figure 2:**
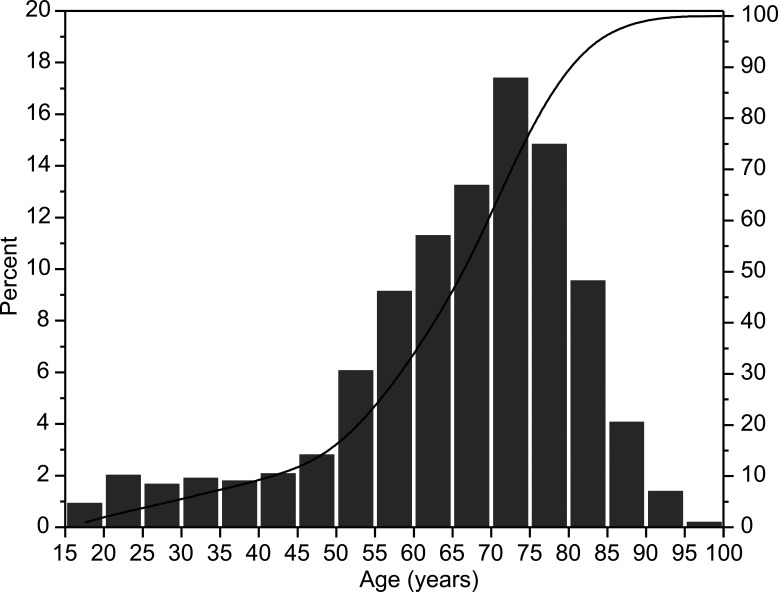
Distribution of hospitalized Croatian patients with herpes zoster in respect to age: distribution by 5-year age-bands (bars) and cumulative distribution (line) (N = 1755).

**Figure 3:**
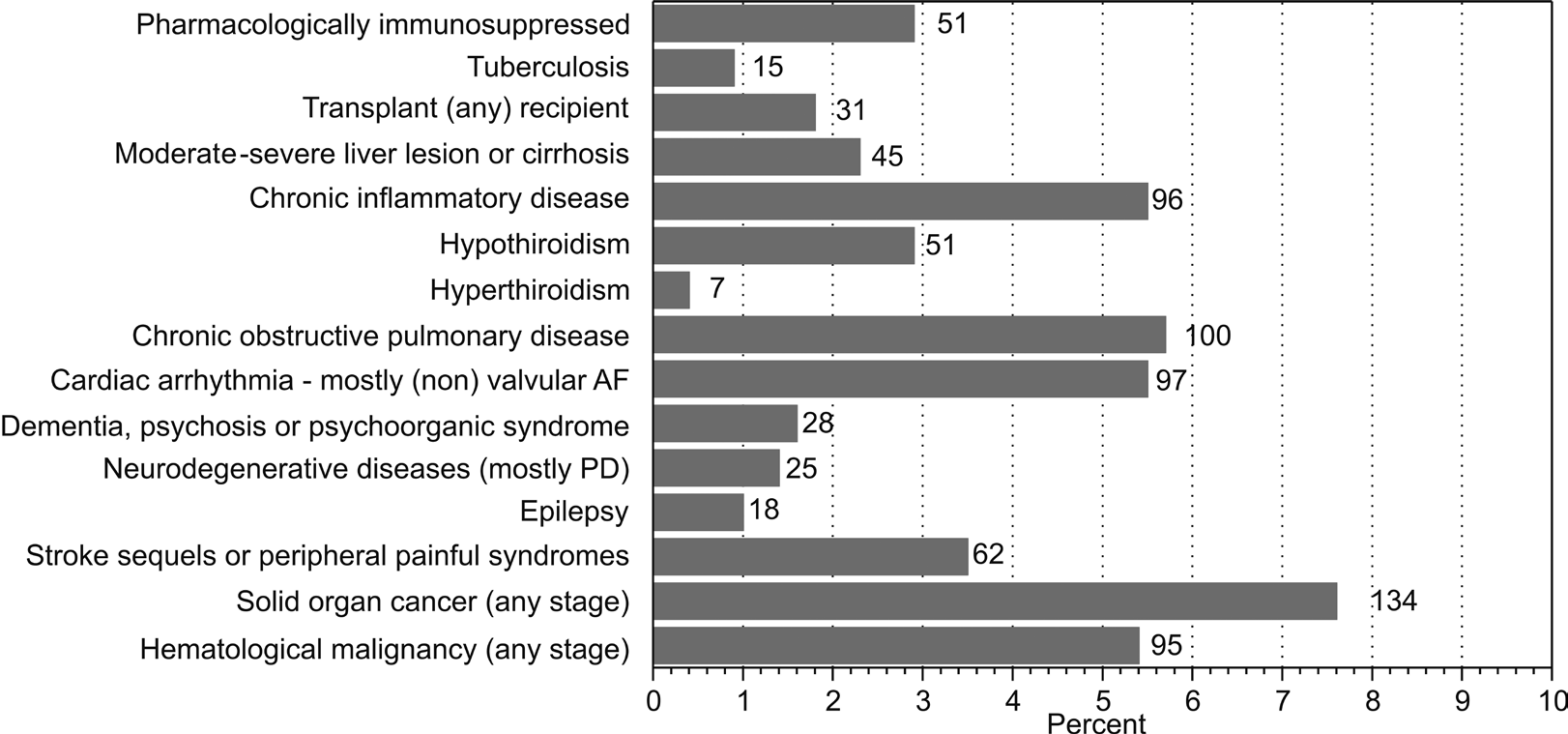
Comorbidities recorded in <10% of the analyzed patients (N = 1755). Inserted are absolute numbers. AF– atrial fibrillation; PD – Parkinson’s disease.

The median age was 70 years (range 17 to 97) (Table 2), and only 20% of patients were younger than 55 years (Figure 2). Age steadily increased over the observed period (Table 2). There was somewhat fewer male than female patients (roughly 45% vs 55%) (Table 2). Overall, 61.6% patients suffered from at least one comorbid condition, and 28.2% had two or more comorbidities (Table 2). Both proportions varied over the observed period, but with an increasing trend over the last several years (Table 2, Figure 1B). The only comorbidities observed in ≥10% of patients were diabetes (14.6%), MACE (24.4%), co-existing infection (12.9%), and cancer (13.0%) (Table 2). The proportions of patients with diabetes, co-existing infection, and cancer varied over the years with no obvious trend, whereas the proportion of patients with MACE steadily decreased from 2004-2006 onward (Figure 1B). A number of comorbidities were observed in <10% of patients, while only 2.9% of the patients where pharmacologically immunosuppressed (Figure 3). Charlson comorbidity index was consistently 3-4 (median) (Table 2). None of the hospitalized patients had an HIV infection.

**Table 2:** Demographics and major comorbidities (in ≥10% of the patients). Data are median (range) or count (%)

Year	N	Age (years)	Men	Any comorbidity	≥2 conditions	Diabetes	MACE*	Coinfection	Any cancer	CCI^✝^
Total	1755	70 (17-97)	742 (42.3)	1075 (61.6)	494 (28.2)	256 (14.6)	428 (24.4)	226 (12.9)	228 (13.0)	3 (0-14)
1996	85	68 (18-92)	28 (32.9)	50 (58.8)	25 (29.4)	6 (7.1)	28 (32.9)	19 (22.3)	7 (8.2)	3 (0-8)
1997	73	67 (25-88)	24 (32.9)	29 (39.7)	15 (20.6)	6 (8.2)	14 (19.2)	12 (16.4)	8 (11.0)	3 (0-11)
1998	76	67 (28-89)	31 (40.8)	41 (53.9)	16 (21.1)	12 (15.8)	17 (22.4)	12 (15.8)	5 (6.6)	3 (0-8)
1999	83	67 (20-93)	27 (32.5)	41 (49.4)	19 (22.9)	13 (15.7)	19 (22.9)	15 (18.1)	9 (10.8)	3 (0-10)
2000	99	68 (22-91)	33 (33.3)	66 (61.6)	24 (24.4)	20 (20.2)	30 (30.3)	16 (16.2)	5 (5.1)	3 (0-10)
2001	86	69 (17-94)	42 (48.8)	52 (60.5)	29 (33.7)	17 (19.8)	32 (37.2)	11 (12.8)	7 (8.1)	3 (0-9)
2002	74	68 (19-89)	37 (50.0)	47 (63.5)	22 (29.7)	10 (13.5)	25 (33.8)	13 (17.6)	10 (13.5)	3.5 (0-10)
2003	85	68 (21-93)	38 (44.7)	62 (73.8)	20 (23.5)	11 (12.9)	33 (38.8)	10 (11.8)	14 (16.5)	4 (0-14)
2004	75	69 (21-96)	34 (45.3)	50 (66.7)	17 (22.7)	9 (12.0)	15 (20.0)	7 (9.3)	15 (20.0)	3 (0-10)
2005	93	68 (19-89)	39 (41.9)	65 (68.9)	41 (44.1)	18 (19.3)	34 (36.6)	8 (8.6)	16 (17.2)	4 (0-11)
2006	93	72 (19-90)	48 (51.6)	62 (66.7)	23 (24.7)	12 (12.9)	31 (33.3)	0	18 (19.4)	4 (0-11)
2007	69	70 (21-93)	30 (43.5)	50 (72.5)	26 (37.7)	17 (24.6)	27 (39.1)	10 (14.5)	8 (11.6)	4 (0-11)
2008	93	70 (19-89)	40 (43.0)	64 (69.6)	31 (33.3)	11 (11.8)	24 (25.8)	12 (12.9)	22 (23.7)	4 (0-10)
2009	73	72 (24-96)	34 (46.6)	44 (60.3)	19 (26.0)	12 (16.4)	12 (16.4)	6 (6.9)	8 (11.0)	3 (0-11)
2010	67	70 (30-91)	29 (43.3)	36 (54.5)	16 (23.9)	12 (17.9)	4 (6.0)	14 (20.9)	6 (9.0)	3 (0-10)
2011	97	66 (17-91)	44 (45.4)	58 (59.8)	29 (29.9)	8 (8.3)	10 (10.3)	14 (14.4)	9 (9.3)	3 (0-10)
2012	87	70 (25-91)	36 (41.4)	46 (54.8)	15 (17.2)	8 (9.2)	9 (10.3)	5 (5.8)	13 (14.9)	3 (0-10)
2013	81	70 (18-91)	34 (42.0)	41 (50.6)	21 (25.9)	10 (12.3)	15 (18.5)	3 (3.7)	4 (4.9)	3 (0-9)
2014	92	72 (27-90)	36 (39.1)	63 (69.2)	32 (34.8)	18 (19.6)	15 (16.3)	20 (21.7)	12 (13.0)	4 (0-11)
2015	85	74 (18-92)	34 (40.0)	54 (64.3)	29 (34.1)	11 (12.9)	17 (20.0)	8 (9.4)	17 (20.0)	3 (0-12)
2016	89	73 (23-97)	44 (49.4)	59 (67.1)	25 (28.1)	15 (16.9)	17 (19.1)	12 (13.5)	15 (16.8)	4 (0-14)

*Major adverse cardiovascular events: history of acute myocardial infarction, any stroke or transitory ischemic attack, chronic heart failure regardless of clinical stage or venous thromboembolism within 6 months.

✝Charlson comorbidity index.

The average hospitalization length decreased, from median 11 to median 9 days (Table 3). Deaths were sporadic (Table 3). The proportion of patients discharged as “cured” varied (Table 3), but there appeared an increasing trend, particularly from 2004-2006 onward (Table 3), which coincided with a decreasing prevalence of patients with a complicated form of the disease (Figure 1A).

**Table 3:** Clinical outcomes. Data are median (range) or count (percent)

Year	N	Hospital stay (days)	Improved	Cured	Unchanged*	Died
Total	1755	10 (2-279)	931 (53.0)	768 (43.8)	40 (2.3)	16 (0.9)
1996	85	11 (5-38)	56 (65.9)	27 (31.8)	2 (2.3)	0
1997	73	12 (4-27)	52 (71.2)	20 (27.4)	1 (1.4)	0
1998	76	11 (4-57)	64 (84.2)	7 (9.2)	4 (5.3)	1 (1.3)
1999	83	11 (6-279)	61 (73.5)	18 (21.7)	3 (3.6)	1 (1.2)
2000	99	11 (7-44)	55 (55.6)	41 (41.4)	1 (1.0)	2 (2.0)
2001	86	10 (4-32)	41 (47.7)	43 (50.0)	1 (1.2)	1 (1.2)
2002	74	10 (5-27)	42 (56.8)	28 (37.8)	2 (2.7)	2 (2.7)
2003	85	9 (4-29)	27 (31.8)	54 (63.5)	1 (1.2)	3 (3.5)
2004	75	9 (4-264)	44 (58.7)	29 (38.7)	1 (1.3)	1 (1.3)
2005	93	10 (5-23)	74 (79.6)	17 (18.3)	2 (2.1)	0
2006	93	9 (5-25)	57 (61.3)	35 (37.6)	1 (1.1)	0
2007	69	9 (2-29)	25 (36.2)	40 (58.0)	3 (4.4)	1 (1.4)
2008	93	9 (5-43)	30 (32.3)	62 (66.7)	1 (1.1)	0
2009	73	9 (2-101)	30 (41.1)	40 (54.8)	2 (2.7)	1 (1.4)
2010	67	9 (3-48)	32 (47.8)	34 (50.8)	1 (1.5)	0
2011	97	9 (5-83)	45 (46.4)	50 (51.5)	2 (2.1)	0
2012	87	9 (4-43)	48 (55.2)	37 (42.5)	2 (2.3)	0
2013	81	9 (2-19)	31 (38.3)	49 (60.5)	0	1 (1.2)
2014	92	9 (4-61)	40 (43.5)	46 (50.0)	5 (5.4)	1 (1.1)
2015	85	9 (3-28)	37 (43.5)	45 (52.9)	2 (2.3)	1 (1.2)
2016	89	9 (4-43)	40 (44.9)	46 (51.7)	3 (3.4)	0

*Refers to patients who were not discharged due to an improved condition but were transferred to other hospitals before relevant improvement/cure was achieved.

### Exploratory analysis

**Figure 4:**
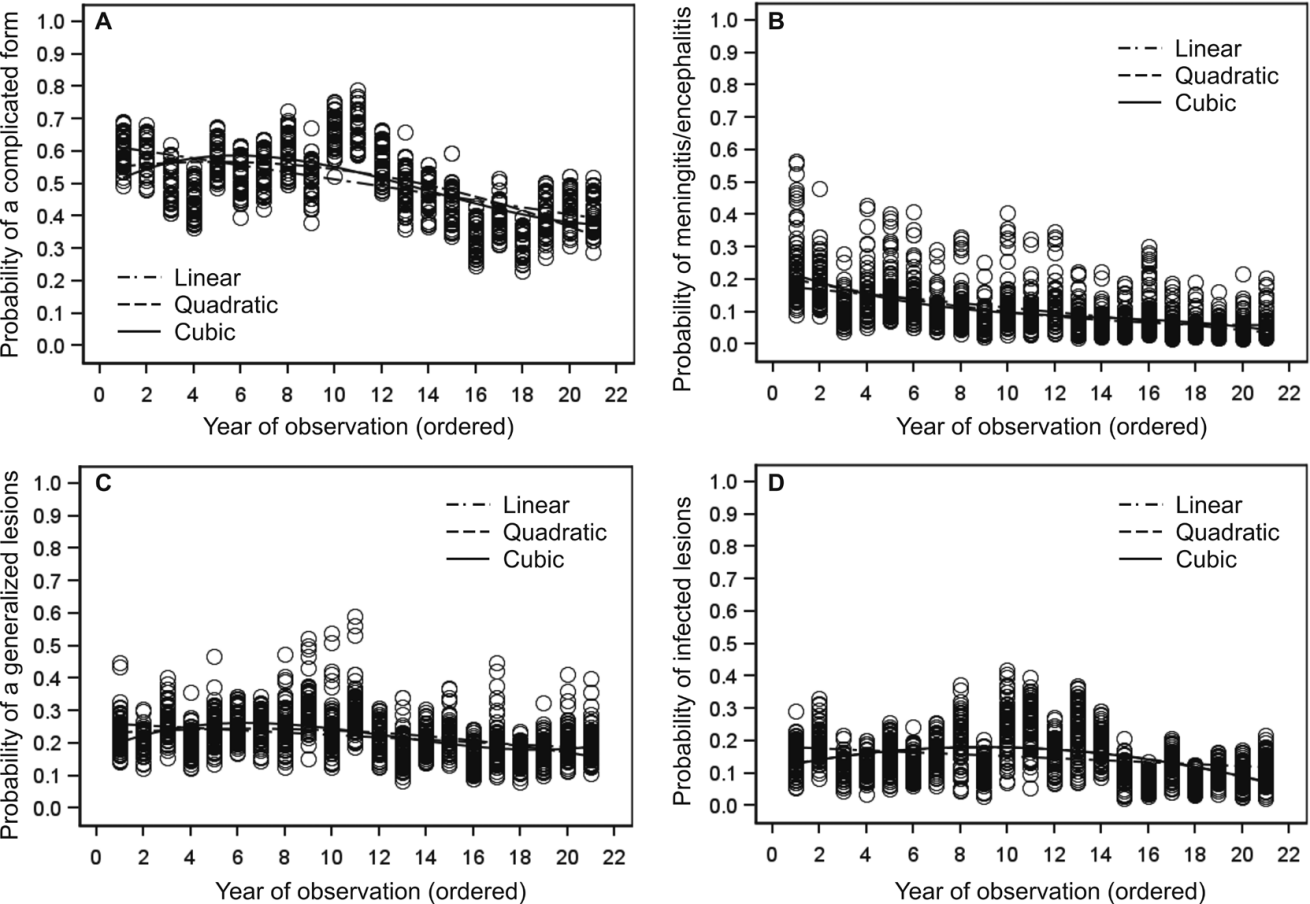
Adjusted probabilities (from models in Table 4) of (**A**) Any complicated form; (**B**) Meningitis/encephalitis; (**C**) Generalized lesions; (**D**) Infected lesions – over the observed period. Fitted lines illustrate linear, quadratic, and cubic trends.

The odds of any complicated form of HZ and the odds of herpetic meningitis/encephalitis decreased over the observed period (linear trend; quadratic and cubic were not significant) (Table 4, Figure 4A and B), while no clear trend was observed regarding generalized or infected skin lesions (Table 4, Figure 4C and D). Older age was associated with a lower odds of herpetic meningitis/encephalitis and with a higher odds of generalized or infected skin lesions (Table 4). Male sex was associated with a higher odds of any complicated form, and pharmacological immunosuppression was associated with a higher odds of generalized lesions (Table 4). No other association was observed between the four outcomes and patient demographics or major comorbidities and pharmacological immunosuppression (Table 4).

**Table 4:** Summary of multivariate* models fitted to probability of (a) any complicated form; (b) herpetic meningitis/encephalitis; (c) generalized skin lesions, and (d) infected herpetic skin lesions. Effects are odds ratios (OR). Observed and multiplicity-adjusted confidence intervals and *P* values are reported

	Outcome: any complicated form				Outcome: herpetic meningitis/encephalitis			
	observed effect		adjusted		observed effect		adjusted	
	OR (95% CI)	P	95% CI	P	OR (95% CI)	P	95% CI	P
Age	1.00 (0.99-1.01)	0.788	0.99-1.01	0.788	0.97 (0.96-0.98)	<0.001	0.95-0.98	<0.001
Men	1.45 (1.19-1.77)	<0.001	1.09-1.93	0.002	1.13 (0.82-1.58)	0.451	0.71-1.81	0.832
Diabetes	0.83 (0.63-1.10)	0.201	0.56-1.24	0.591	1.04 (0.65-1.67)	0.866	0.53-2.05	0.981
Malignant disease	0.80 (0.59-1.08)	0.114	0.52-1.23	0.591	0.52 (0.28-0.97)	0.039	0.21-1.26	0.211
Coexisting infection	0.91 (0.67-1.22)	0.519	0.59-1.38	0.772	1.39 (0.88-2.22)	0.162	0.72-2.70	0.505
Immunosuppressed	1.57 (0.85-2.91)	0.151	0.65-3.78	0.591	1.11 (0.36-3.37)	0.859	0.23-5.39	0.981
Major adverse cardiovascular events	1.12 (0.87-1.43)	0.377	0.79-1.59	0.750	1.41 (0.93-2.13)	0.107	0.78-5.39	0.431
Observed years	0.96 (0.93-0.98)	0.011	0.92-1.00	0.050	0.94 (0.90-0.97)	0.002	0.89-0.98	0.014

	Outcome: generalized skin lesions				Outcome: infected herpetic skin lesions			
	observed effect		adjusted		observed effect		adjusted	
	OR (95% CI)	P	95% CI	P	OR (95% CI)	P	95% CI	P
Age	1.01 (1.00-1.02)	0.002	1.00-1.03	0.017	1.02 (1.01-1.03)	<0.001	1.00-1.03	0.006
Men	1.31 (1.04-1.66)	0.024	0.94-1.83	0.136	1.40 (1.06-1.85)	0.016	0.95-2.08	0.109
Diabetes	0.76 (0.54-1.07)	0.115	0.47-1.23	0.386	1.12 (0.77-1.62)	0.541	0.67-1.89	0.789
Malignant disease	1.12 (0.79-1.60)	0.521	0.68-1.85	0.762	0.99 (0.65-1.51)	0.966	0.54-1.81	0.966
Coexisting infection	0.77 (0.53-1.11)	0.164	0.45-1.30	0.417	0.77 (0.49-1.20)	0.244	0.41-1.44	0.676
Immunosuppressed	2.36 (1.26-4.44)	0.008	0.96-5.79	0.052	0.35 (0.10-1.20)	0.094	0.06-1.99	0.444
Major adverse cardiovascular events	0.91 (0.68-1.21)	0.513	0.60-1.37	0.762	1.18 (0.85-1.63)	0.322	0.74-1.86	0.692
Observed years	0.97 (0.94-1.00)	0.052	0.94-1.01	0.236	0.97 (0.93-1.01)	0.152	0.91-1.03	0.560

*See Patients and Methods for the rationale of the fitted models and multiplicity adjustment.

## Discussion

Overall, our observations suggest: a) there is a tendency of hospitalizing older patients even with non-complicated disease forms, but on the account of comorbidities and/or older age itself; b) younger patients seemingly are hospitalized only when serious complicated forms are present – eg, meningitis/encephalitis – that require other supportive measures. Less severe complicated forms (eg, infected lesions, generalized lesions) and non-complicated presentations do not seem to lead to a hospitalization in younger patients (likely also less burdened with comorbidities). The observed reduced hospital stay over the years indirectly supports such a view: when patients are hospitalized with milder disease forms (on the account of age or some comorbidity), resolution is likely to occur earlier, resulting in a shorter hospital stay. Next, we observed no association between major comorbidities (diabetes, malignancy, co-infection, MACE) and presentation with a complicated form of the disease (overall and by specific form). However, this could be a form of “confounding-by-indication” since the cohort comprised only hospitalized patients: complicated forms might have been admitted regardless of comorbidity, while uncomplicated forms could have been admitted on the account of comorbidity. Having in mind this limitation, we observed an independent association between pharmacological immunosuppression and the presence of generalized skin lesions. Although confounding (as explained) cannot be excluded, it appears reasonable to consider this observation as supportive to a conclusion about a causal relationship between immunosuppression and this specific form of complicated HZ.

The present report is the largest described cohort of HZ patients from Croatia. Its limitations are severalfold: a) it reports only on hospitalized patients, hence provides no insight into clinical presentation of HZ patients in general; b) it is single-centered; c) it is focused on primary clinical presentation, not on disease sequels, eg, post-herpetic neuralgia. However, the data were collected in the largest national center for infectious diseases with the catchment area corresponding to around 25% of the adult Croatian population, hence the observed patterns are likely to reflect trends on the national level. Recent reports from several European countries (17-19) demonstrate increasing cost burden of HZ hospital treatments – even when only primary presentations are considered. This is a result of the increasing incidence of HZ related to aging population and, hence, a higher prevalence of predisposing comorbidities (17-19). Considering the progressively aging population, a need has been recognized for measures that could reduce the overall disease burden (17-19). A possibility of vaccination of older adults in order to prevent HZ has attracted much attention, since the available vaccines considerably reduce disease incidence in otherwise healthy older individuals (20). However, feasibility of any general health care measure in the population depends not only on the effectiveness of available means and disease incidence, but also on disease characteristics in particular communities. In this respect, the present study provides the first informative data on clinical characteristics of adults and adolescents hospitalized for HZ in Croatia. Two Italian studies based on reviews of hospital discharge forms report on similar outcomes for the period 1999-2005 (21) and 2001-2013 (22) and provide a relevant reference point for the present observations. Overall, the present cohort shares certain common characteristics with the Italian nationwide cohort 1999-2005 (21) and, particularly, with the more recent cohort from three Italian regions (22): a slight predominance of women among hospitalized patients (approximately 55% vs 45%), around 50%-55% of hospitalized patients with uncomplicated disease, similar (around 10%) (21) or somewhat higher (22) proportion of patients with neurological complications, and a low prevalence of herpetic keratitis, Ramsey-Hunt syndrome, or visceral dissemination, with all-cause mortality roughly around 1% (21,22). Further similarities include a wide range of patient age (from adolescents to very old people), but with a clear predominance of the elderly, and similar average hospital stay during the index hospitalization (21). The prevalence of comorbidities considered relevant for the occurrence of HZ or considered as poor prognostic factors (eg, malignancy, diabetes, chronic obstructive pulmonary disease, autoimmune diseases) was also similar (21,22).

Unfortunately, the present data cannot provide an insight into annual hospitalization rates. A rough approximation based on more or less stable absolute number of hospitalized patients over the observed period and assuming a stable population would suggest that it has remained constant, but this is only an assumption. However, several trends were apparent. Over the observed period the age of hospitalized patients steadily increased, which coincided with a decreasing proportion of complicated disease forms and particularly meningitis/encephalitis, and with an increasing proportion of patients with at least one comorbid condition (over the last several years of observation).

In conclusion, clinical characteristics of Croatian patients hospitalized for HZ appear similar to those of patients in developed European countries.
